# Exosomes derived from bone marrow mesenchymal stem cells ameliorate chemotherapeutically induced damage in rats’ parotid salivary gland

**DOI:** 10.1007/s10006-025-01331-9

**Published:** 2025-01-17

**Authors:** Ahmed Zakaria, Nessma Sultan, Nesreen Nabil, Mahitabe Elgamily

**Affiliations:** 1 Egyptian Ministry of Health, Mansoura, Egypt; 2 Oral Biology, Faculty of Dentistry, Mansoura University, Mansoura, Egypt; 3 Oral Biology and Dental Morphology, Faculty of Dentistry, Mansoura National University, Gamasa, Egypt; 4 Oral Biology Department, Faculty of Dentistry, Mansoura University, Mansoura, Egypt; 5 Oral Biology Department, Faculty of Dentistry, Egyptian Russian University, Cairo, Egypt

**Keywords:** Parotid gland, Chemotherapy, Bone marrow, Stem cells, Exosomes, Malondialdehyde

## Abstract

**Objective:**

A nanometer-sized vesicles originating from bone marrow mesenchymal stem cells (BMMSCs), called exosomes, have been extensively recognized. This study defines the impact of BMMSCs and their derived exosomes on proliferation, apoptosis and oxidative stress (OS) levels of CP-induced parotid salivary gland damage.

**Methods:**

BMMSCs were isolated from the tibia of four white albino rats and further characterized by flowcytometric analysis. BMMSCs-derived exosomes were harvested and underwent characterization using transmission electron microscopy (TEM), western blot analysis and BCA assay. Fifty-six healthy white albino male rats weighting from 200 to 250 g were allocated into 4 groups (*n* = 14); Group I, rats received phosphate buffered saline (PBS), group II, rats were intraperitoneally injected with CP, group III& IV received CP and after 3 days they were intravenously injected with either BMMSCs (group III) or BMMSCs-exosomes (group IV). Histological, and immunohistochemical studies using proliferating cell nuclear antigen (PCNA) were done after 7 and 14 days. The OS was measured using malondialdehyde (MDA) and apoptosis was measured by annexin V-FITC/PI.

**Results:**

BMMSCs and exosomes treated groups showed better histological features approximating the normal architecture of the control group. The percentage of PCNA positively stained cells were significantly higher in the exosomes treated group in comparison to all other groups. MDA assay test revealed that the exosomes were able to reduce the OS when compared to the cell-based therapy using BMMSCs. Annexin V revealed that BMMSCs-exosomes significantly reduced the percentage of apoptotic cells compared to other treated groups.

**Conclusions:**

BMMSCs-exosomes could improve the CP-induced cytotoxicity in rats’ parotid salivary gland.

## Introduction

The acinar-ductal unit of salivary gland has a key role in the production and secretion of saliva. Saliva is important for digestion of foods, taste, and lubrication, along with oral homeostasis and immune response [1]. Radiation therapy and chemotherapy can cause secretory dysfunction resulting in pathologic outcomes including difficult swallowing, taste loss, slurred speech, caries, and candidal infection [2–6].

One of the most extensively utilized antineoplastic agents in treating a range of head and neck tumours is cisplatin (CP). CP has the capacity to cross-link with purine bases on DNA to form adducts. In addition to causing DNA damage, this crosslinking disrupts DNA repair pathways, stops the cell cycle in G2 phase, and ultimately triggers apoptosis of tumour cells. In addition, the disruption of mitochondria which leads to a decrease in ATPase activity, and alterations to cellular transport systems are linked to the cytotoxicity of CP on cancer cells. CP not only ruins DNA but also releases reactive oxygen species, which are lethal to cells. Even though CP has potent chemotherapeutic actions, some unfavourable side effects could result from its cytotoxic effects. Also, CP is not selective for tumour cells because it also influences healthy cells [7, 8].

Salivary regenerative methods are focused on cell-based approaches, which require the identification of potential stem/progenitor cells for substitution of destructed acinar-ductal unit. Precious reports revealed the presence of stem cells in salivary glands of mice, rats, and humans; however, they are scarce [9–11]. So, the use of a more abundant source of stem cells, like bone marrow mesenchymal stem cells (BMMSCs), may overcome such scarcity of stem cells in salivary glands. BMMSCs have the potential of differentiation into multiple cell types. BMMSCs have also been extensively investigated in the in vitro and in vivo studies because they have anti-inflammatory activities, low-immunogenic, and are able to repair damaged cells [12–18].

Recently, the paracrine factors released from BMMSCs has been investigated and allowed tissue regeneration through the modulation of the immune reactions, alleviating inflammatory response and fibrotic changes, inducing angiogenesis and neurogenesis, and inhibiting apoptosis [19, 20]. This has led to the development of several cell-free treatments over the previous few years. These include cell extract therapy, conditioned medium therapy, and other therapies (e.g., extra-cellular vesicle therapy). Exosomes are extra-cellular vesicles with diameters ranging between 30 and 100 nm. Exosomes have been found to have a role in cell-to-cell communication through the transportation of different proteins, microRNAs and mRNAs. The therapeutic effect of these extra-cellular vesicles for regenerative medicine is still ongoing research. Since parent cell selection enhancing specificity, preventing immune disorders or rejection observed in cell therapies, exosomes continue to improve in regenerative medicine. BMMSCs-exosomes has been considered promising for treating many diseases due to their abundant content and their potential advantage as a carrier [21, 22].

Most of studies on BMMSCs therapy for salivary glands focused mainly on the detrimental effects of radiotherapy on submandibular glands [23–25]; few examined the effect of chemotherapeutic drugs on salivary glands, and limited studies on parotid glands, were found to date. Additionally, to the best of our knowledge, no research found testing the potential therapeutic role of BMMSCs and their exosomes on proliferative index, apoptosis and OS on the parotid salivary glands received CP. Thus, the current study aimed at investigating whether BMMSCs-exosomes can restore the structure of salivary gland tissue after its intravenous injection in albino rat model subjected to CP-induced damage of parotid glands. Moreover, the proliferation rates, apoptosis and OS levels were measured in salivary cells to discover the possible mechanism of regeneration after BMMSCs transplantation.

## Materials and methods

The ethical committee of Faculty of Dentistry, Mansoura University approved this study; code no: MU-ACUC (DENT.MS.22.11.3) and all procedures were performed according to ARRIVE guidelines (Animal Research: Reporting In Vivo Experiments) for reporting animal research.

### Animals and study design

In this study, sixty Sprague-Dawley albino male rats weighting 200–250 g (aged 5–6 months) were utilized. BMMSCs were isolated from four rats, and the remaining were kept in cages in the animal house in faculty of medicine, Ain Shams Research Institute (MSRI). Animals were acclimatized by getting free access to food and water, they were fed standard pellet of diet and tap water throughout the study period. The room temperature was about 22–24 ^o^C and animals were exposed to 12:12 h light/dark cycles for at least 14 days before starting the experiment. They were then divided into 4 groups (*n* = 14):

Group I: rats received 0.5 ml of PBS injected into tail veins and considered as negative control. Group II: rats received an intraperitoneal injection of CP (single dose of 5 mg/kg) [26] and considered a positive control group in which the animals did not receive any further treatment. In both group III & IV, the rats received single intra-peritoneal CP injection (5 mg/kg) and after 3 days they received intravenous injection into the tail vein with either BMMSCs (2 × 10^6^ cells) suspended in 0.5 ml of PBS (group III) [27, 28] or BMMSCs-exosomes (100 µg/kg/dose suspended in 0.2 ml PBS) (group IV) [29].

### Rat BMMSCs harvesting

Steps have been performed at central lab of stem cells and biomaterials applied research, Faculty of Dentistry, Ain Shams University. Briefly, four albino rats were used to obtain intact tibiae, bones were transferred to laminar flow cabinet (Thermo scientific™ Thermo Safety Bench, Waltham, USA) in transfer media; Dulbecco’s modified eagles’ medium (DMED; Gibco, Thermo-scientific, Germany) supplemented with penicillin, streptomycin, L-glutamine (100 µg/ml, 100 µg/ml and 2 mM, respectively) without fetal bovine serum (FBS) (Life Science, UK). Inside laminar flow cabinet, heads of bones were removed using a sterile scissor. Bone marrow was aspirated with sterile needle and re-suspended in DMEM culture media in T75 flask.

The culture flasks underwent incubation at 37 ^o^c in 5% CO2 and 95% air by volume in an incubator (Thermo scientific HERA cell VIOS 160i CO2 incubator with IR sensor, Waltham, USA). Daily examination of the cultured cells was done by using an inverted light microscope (LeicaDMi8-Automated Inverted fluorescence microscope, Wetzlar, Germany) to follow up cell growth and to detect any bacterial or fungal growth.

After 48 h, a sterile pipette was used to aspirate and remove the supernatant. After that, the adherent cells were washed 2-times using a sterile PBS and 10 ml of fresh complete media (DMED + 10% FBS) was added to the flask. After 2–3 days, the media was changed. Cells were sub-cultured when they became approximately 70–80% confluent. After washing the cells with PBS, 1 ml of 0.05% trypsin/ Ethylenediaminetetraacetic acid (EDTA-Lonza^®^) was added to the flask with monitoring of the cell detachment for 2–5 min, then 5 ml of complete medium (DMEM + 10% FBS) was added to neutralize the trypsin; and the trypsin/medium/cell suspension was collected in a tube and centrifuged (Thermo scientific Heraeus Fresco 21 microcentrifuge-75002425, Waltham, USA) at 600 rpm for 5 min. Discarding of the supernatant was done, and pellets re-suspended in pre-warmed complete medium in T 75 flask. BMMSCs from 2nd to 5th passages were utilized in this experiment.

### Cell counting

A hemocytometer was utilized to count the cells. Briefly, 25 µl of cell suspension was added to 25 µl of trypan blue solution in buffer and then added to the hemocytometer. The chamber was placed in the Invitrogen™ Countess™ Automated Cell Counter (Thermo scientific countess 3, Waltham, USA).

### Characterization of BMMSCs

The cultured BMMSCs were characterized using Fluorescent Activated Cell Sorting (FACS). Different fluorescently-labelled monoclonal antibodies (eBioscience, company) were used to stain the cells. Briefly, 5 × 10^5^ cells (in 100 µl PBS/0.5% BSA/2 mmol/EDTA) underwent mixing with 10 µl of fluorescently-labelled antibodies and then underwent incubation in the dark at 2∼8^o^C for half an hour. Washing was done twice using PBS containing 2% BSA, and pellets were re-suspended in PBS and analysed by EPICS-XL flow cytometer (Coulter, Miami, Fl, US). The positivity of cluster of differentiation CD105, CD90 and negativity of CD34 were measured [30–31].

### BMMSCs-Exosomes isolation

Isolation has been performed at Nawah Scientific Research Centre. BMMSCs at 3rd passage were cultured till reaching a confluence of 70–80% then changing the complete media and washing the flasks using PBS. Cells were left in serum free media low glucose DMEM and were incubated in CO2 incubator for 2 days to produce exosomes. Then, centrifugation of the media was done at 2500 rpm for 10 min to get rid of any cells. Then, filtration using 0.45 μm cell sieve, then 0.22 μm (CA 0.22 μm membrane solutions, chongqing, china) to eliminate any debris. The supernatant underwent ultracentrifugation (Beckman Coulter Optima™ L-80XP, California, USA) at 90.174 × 10^5^ rpm at 4 °C for 90 min with a type 50.2 Ti rotor (k-factor: 157.7) to pellet exosomes. The supernatant was discarded, and crude exosome pellet was re-suspended in one millilitre of PBS and pooled. Another cycle of ultracentrifugation was carried out, and the resultant exosome-containing pellets were re-suspended [32].

### Characterization of exosomes

#### Characterization by TEM

For fixation, exosomes were immersed with 2.5% glutaraldehyde for 120 min. Washing of exosomes was done then they underwent ultra-centrifugation and suspension in 100 µL human serum albumin. Twenty microliters of exosomes were loaded onto a formvar/carbon coated grid, negatively stained with 3% phosphotungstic acid solution for 1 min. TEM was used for characterization of exosomes (HITACHI, H-7650, Japan) [33].

#### Western blotting of BMMSCs-exosomes

The antibodies used were antigen affinity-purified polyclonal sheep IgG anti-rabbit CD81 (Catalog no. 0349509; BioLegend, San Diego, California, US) and antigen affinity-purified polyclonal IgG anti-rabbit CD83 (Catalog no. MBS127731, My BioSource, Inc., San Diego, California, US). Proteins were extracted from the exosomes using radio-immuno-precipitation buffer’s composition. 20 nano-grams of proteins were loaded and separated by sodium dodecyl sulphate–polyacrylamide gel electrophoresis on 4 ~ 20% polyacrylamide gradient gels. Following incubation at room temperature in 5% non-fat dry milk, Tris-hydrochloride, 0.1% Tween 20 for 60 min, primary antibodies (1:500 dilution factor) were added and incubated overnight. HRP conjugated secondary antibody (Novus Biologicals) solution was incubated against the blotted protein for 120 min. After washing with 1 X TBS-T for 6 times, densitometric evaluation of immunoblots was performed for quantification of CD83 and CD81 against β-actin using image analysis software on the Chemi Doc MP imaging system (Bio-Rad, version 3) [34].

#### Measuring protein concentration of exosomes using BCA assay

Following the manufacturer’s instructions, the BCA Protein Assay Kit (Novagen, Darmstadt, Germany) was used to measure the amount of exosome protein. The BCA solution contained 4% cupric saltate, and the reagent ratio was 2:100. 150µL of the standard and the sample replication were mixed with 150µL of the working agent. After 30 s of shaking the plates to ensure a complete mixing, the plates were incubated for 2 h at 37 °C. A plate reader was used to measure the absorbance at or close to 562 nm after the plates had cooled to room temperature.The standard curve was generated by graphing the mean blank-corrected reading at 562 nm for each BSA standard against its concentration in µg/mL. The protein concentration was determined using the standard curve, with findings expressed in µg/mL [35].

#### Animal sacrificing

**Fig. 1:**
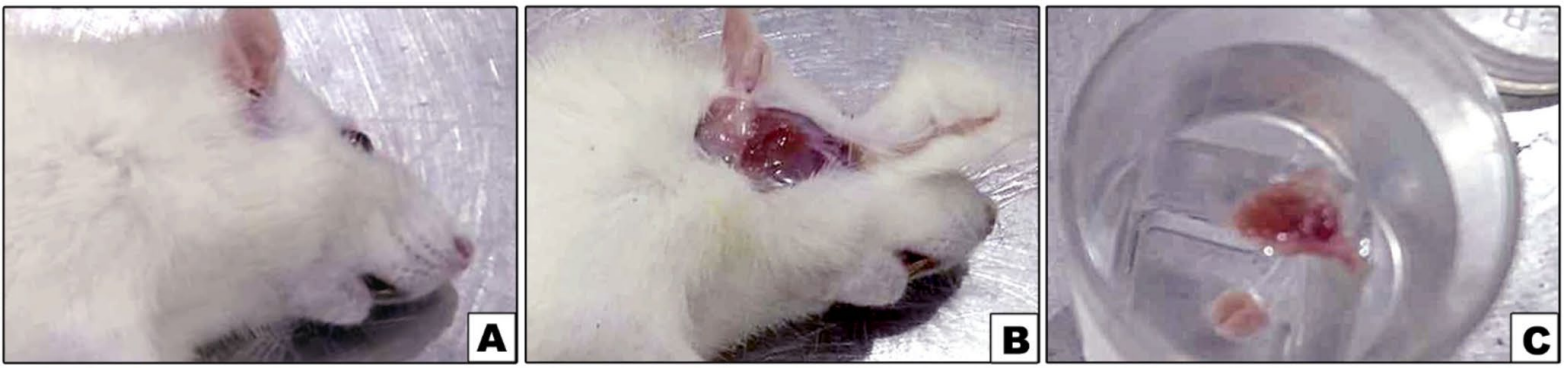
Photographs **A**,** B** and **C** showing the surgical procedure of parotid gland isolation where **B** showing the parotid gland exposure and **C** showing the isolated glands

Rats were scarified using overdose halothane anaesthesia, at 7 and 14 days after injection of CP: 7 rats/ time point. The animal was fixed on the operating board with its head and neck stretched using a tooth holder, we performed a midline skin incision along the rat’s neck using a scalpel. This incision started above the suprasternal notch up to the point of the lower jaw, and extended laterally from the suprasternal notch to the right shoulder and left shoulder. The Each parotid gland was collected after removal of hair and skin over them (Fig. 1), then they were processed for further examination. From each numbered animal, both parotid glands were dissected so 14 parotid glands were obtained in each group/time point. The right sided parotid glands were used for histological analysis while the left sided was used for measuring lipid peroxidation and apoptosis using MDA assay and Annexin V assay, respectively. After scarification and sample collection, the rats were wrapped in plastic bags and carefully transported to be burnt in an incinerator [36].

### Histological analysis

#### Tissue processing

For haematoxylin and eosin analysis and immunohistochemistry, specimens of the right-side gland were immersed in 10% formaldehyde diluted with PBS for fixation. They underwent processing using an automatic tissue processor. They were then embedded with paraffin blocks and sectioned into 4 μm sections. For each histologic block, we prepared multiple sections.

#### Haematoxylin and eosin staining (H&E)

Sections were deparaffinised with xylol, rehydrated with ethanol and stained with hematoxylin. They were then washed with PBS and stained with eosin [37].

#### Immunohistochemical staining (IHC)

Sections were mounted on silane-coated microscope slides, deparaffinised with xylol and rehydrated. Endogenous peroxidase was blocked with hydrogen peroxide at 3% and antigen retrieval was performed at high temperature with citrate buffer 0.01 M (pH 6.0). After that, slides were incubated with the primary monoclonal PCNA antibodies (1:50) (Abcam, Cambridge, UK) and the secondary biotinylated antibodies followed by Streptoavidin-Biotin Peroxidase complex. Development with chromogen substrate diaminobenzidine was performed and slides were counter-stained with Harris haematoxylin. A non-immune serum was used to prepare negative controls. Positive reaction is indicated by brownish staining of nuclei [38].

#### Measurement of lipid peroxidation by malondialdehyde test (MDA)

Sample homogenates from the left-side glands were formed in 5–10 ml cold buffer (50 mM potassium phosphate, pH 7.5. 1 mM EDTA). MDA levels were quantified with thiobarbituric methods (TBARS). The tissue homogenate reacted with TBA reagent containing 0.375% TBA, 15% trichloroacetic acid and 0.25 N HCl. Boiling of samples was done over 10 min, then samples were cooled and underwent centrifugation. The absorbance of supernatants was measured by spectrophotometry at 532 nm. MDA levels (expressed in nmol/g tissue) were calculated on the calibration curve that was prepared utilizing a 1,3,3,3 tetraethoxypropane (ACROS Organics, New Jersey, US) [39–40].

#### Measurement of apoptosis by annexin V/PI

Annexin V kit (Cat. No.556547BD pharmingen FITC apoptosis) was used for the flow cytometric analysis of apoptosis. Briefly, 100 µl of cell suspension from both control and treated parotid salivary gland were collected and were washed with 1 ml PBS in a centrifuge tube followed by spinning at 1800 rpm for 5 min. The supernatant was then removed and the pellets of cells were stained with 5 µl of annexin V-FITC and 5 µl of propidium iodide (PI) in 100 ml of 1x binding buffer. The samples were kept in dark and incubation was done for 15 min. After incubation, cells were ready for acquired on flow cytometry accuri C6 becton dikinson. The percentage of cells analysed by accuri C6 software which represent the quadrant dot plot to perform the four phases of viable, early and late apoptosis and finally necrosis in each group [41].

#### Image analysis

For control and experimental groups, seven slides were prepared at each time point. The slides were divided into four quadrants and one photo was captured from each quadrant at a magnification 100×. Then, 28 images per group were used in analysis. The number of positively expressed cells by PCNA was counted using a computer (Intel^®^ Core I7^®^) and VideoTest Morphology^®^ software (Russia). Images utilized in analysis were stained with PCNA where the positive reaction was brownish in colour.

#### Statistical analysis

Numerical data were tested for normality by testing data distribution, calculation of means and medians and applying Kolmogorov-Smirnov and Shapiro-Wilk tests. Data were parametric and were expressed as means and SDs. Two-way ANOVA was utilized for studying the effect of different variables and their interaction. The main and simple effects were compared using pairwise t-tests with Bonferroni correction. The significance of a result was judged at *P* ≤ 0.05. The IBM^®^ SPSS^®^Statistics Version 26 for Windows was utilized to analyze data. The G*Power version: 3.1.9.7 was used to calculate sample size based on the results of an earlier study [42]. A power analysis was designed to have sufficient power to apply a two-sided statistical test to reject the null hypothesis that no difference does exist among groups. Alpha value was set at (0.05) and beta value was set at (0.2), i.e. power = 80% and an effect size (d) of (0.47) calculated based on an earlier study [43]. The calculated sample size was 56 (14 rats per group), to detect the difference between groups regarding PCNA Area%, MDA (ng/ml) and Annexin V.

## Results

**Fig. 2:**
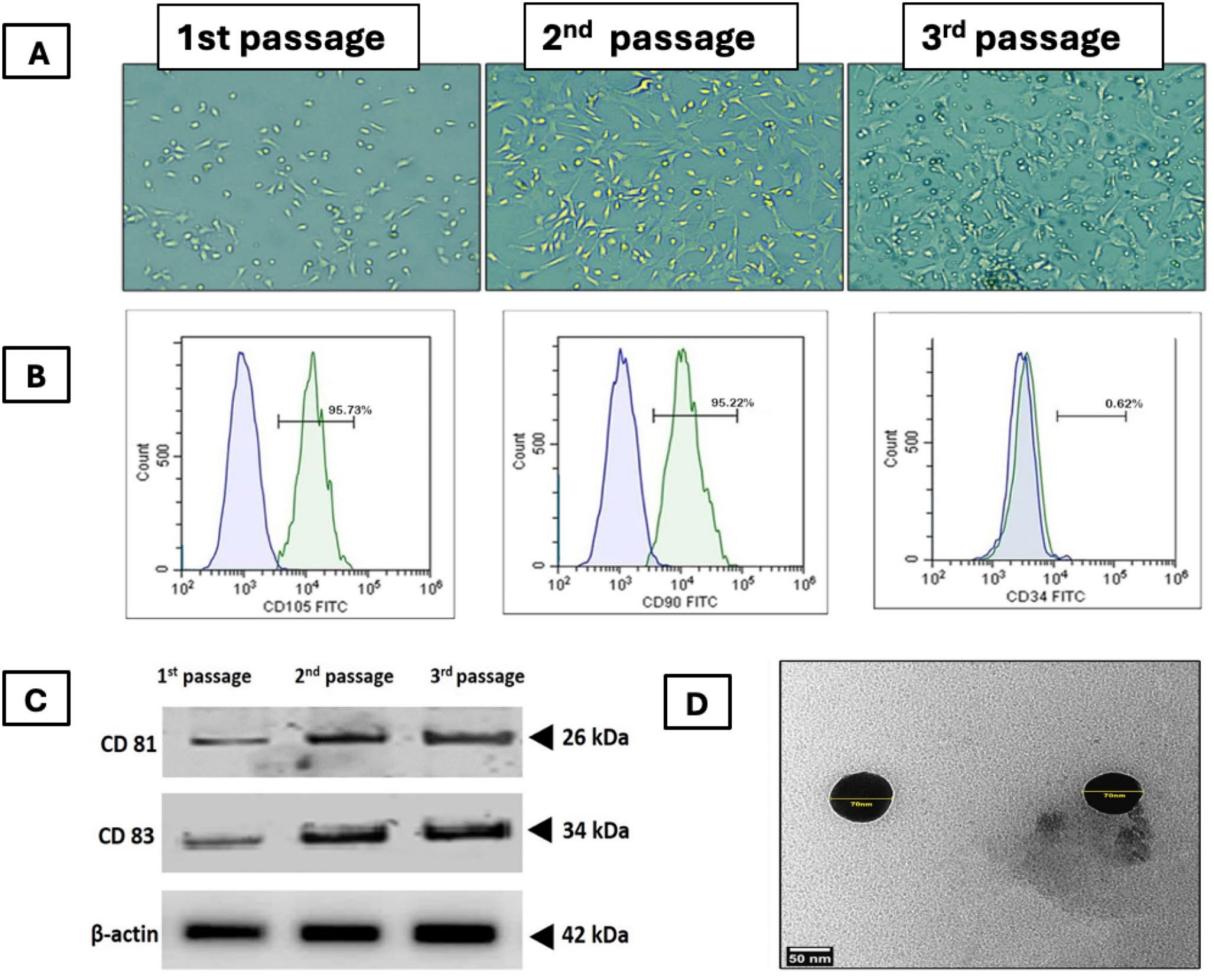
Characterization of BMMSCs and their derived exosomes. (**A**) phase contrast microscopic images showing BMMSCs at different passages, (**B**) Flowcytometric characterization of BMMSCs. (**C**) Western blotting of BMMSCs-exosomes for common markers CD83 and CD81 where, 1^st^ passage unfractionated material and 2^nd^ passage corresponds to the first ultracentrifugation and 3^rd^ passage corresponds to the second ultracentrifugation. (**D**) TEM image showing nanosized vesicles

Characterization of BMMSCs and their derived exosomes (Fig. 2) Cells were identified as MSCs depending upon on their morphological features. They were spindle-shaped after 3rd passage. Flow cytometry indicated that the cells showed positivity for CD90 (95.22%) and CD105 (95.73%), but negativity for CD34 (0.62%). This confirmed the stemness of MSCs. (Fig. 2A& B). Western blotting of BMMSCs-exosomes for CD83 and CD81 was positive (Fig. 2C), TEM image of BMMSCs-derived exosomes showed nano vesicles of size range 70 nm in diameter (Fig. 2D). BCA protein assay was used to determine the overall exosome concentration was determined by quantifying protein concentration from intact exosomes. The concentration of exosome proteins was 974 µg/ml.

### Histological results

There were no deaths or complications in rats reported in the study. After 14 days, there was no indications of necrosis, haematoma, or infections.

#### Haematoxylin and Eosin staining (Fig. 3)

In the negative control group; parotid salivary glands demonstrated the normal histologic and architectural features. Glands consist of lobes and lobules with connective tissue septa separating between them. Each lobule contains spherical serous acini with pyramidal cells surrounding narrow lumen and ducts in between the acini.

**Fig. 3:**
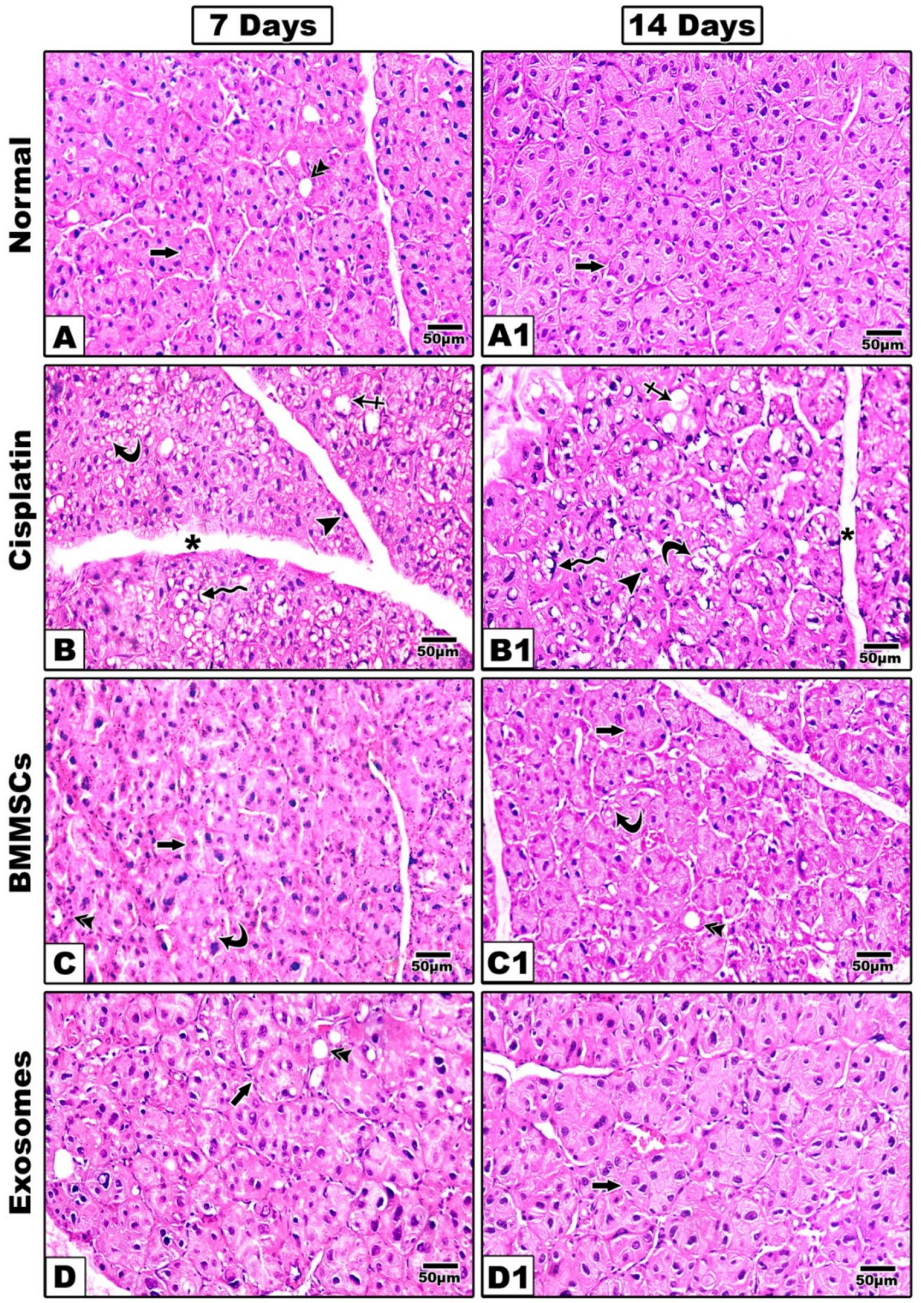
Representative histological images of (H&E) are shown at 7- and 14-days post-treatment. The group received CP (**B, B1**) shows remarkable structural alterations in the acini and ducts with multiple intracytoplasmic vacuoles **(curved arrow)** and ballooning of the acini **(crossed arrow)**. The nuclei were irregular and different in shape and size and some nuclei showed also a signet ring appearance **(zigzag arrow)** other showing hyperchromatic nuclei **(arrow head)**. With noticeable widening of connective tissue septa **(asterisk)**. In BMMSCs treated group (**C, C1**); The acinar outline was still irregular (**arrow**) and acinar cells showed mitotic figures, but intracytoplasmic vacuoles were still detected at 7 days **(curved arrow)** however at 14 days sections showed improved arrangement of acinar and ductal cells with marked decrease in intracytoplasmic vacuoles. Groups treated with BMMSCs-exosomes (**D, D1**) revealed that the acinar **(arrow)**, and ductal outline **(double arrow)** began to regain their normal architecture, the acinar cells showed mitotic figures noteworthy that intracytoplasmic vacuoles were still present although they were decreased in number sections showed well-arranged acini and ducts with almost normal appearance and marked reduction in the intracytoplasmic vacuoles. Scale 100x

##### After 7 days

In the positive control group received CP; remarkable structural alterations in the acini and ducts in comparison to those of the negative control group. Sections demonstrated serous secretory acini with poorly defined outline and many intra-cytoplasmic vacuoles, hydropic degeneration and ballooning of the acini was also evident (Fig. 3B). The nuclei were irregular and different in shape and size. Variable signs of nuclear apoptosis were also detected as irregular or hyperchromatic shrunken nuclei compressed by variable-sized cytoplasmic vacuoles, some nuclei showed also a signet ring appearance. Ducts appeared distorted with focal loss of their lining epithelium and basement membrane. Connective tissue septa were widened. In BMMSCs treated group; The acinar outline was still irregular and acinar cells showed mitotic figures, but intracytoplasmic vacuoles were still detected, the ductal outline began to regain its normal architecture (Fig. 3C). Groups treated with BMMSCs-exosomes revealed that the acinar and ductal outline began to regain their normal architecture, the acinar cells showed mitotic figures noteworthy that intracytoplasmic vacuoles were still present although they were decreased in number (Fig. 3D).

##### After 14 days

In CP treated group (Fig. 3B1), the signs of cellular damage were still present with complete loss of some acini. Abnormal morphology of many nuclei with chromatin condensation or pyknosis and complete degeneration of others. Vacuolization, aggravation of ballooning degeneration and fibrosis of the connective tissue septa were also evident. In BMMSCs treated group (Fig. 3C1); sections showed improved arrangement of acinar and ductal cells with marked decrease in intracytoplasmic vacuoles while in exosomes treated group; sections showed well-arranged acini and ducts with almost normal appearance and marked reduction in the intracytoplasmic vacuoles (Fig. 3D1).

### Immunohistochemical staining (Fig. 4)

The immunohistochemical positive results of the PCNA were detected as brown colour staining the nucleus. In the negative control group, sections demonstrated positive immune labelled cells, with a random distribution in salivary gland revealing normal mitotic activity (after 7 days: 3.93±0.27, after 14 days: 4.51±0.20), while in CP group, the number of positively stained cells showed a significant reduction in comparison to all other groups either after 7 days (0.66±0.03) or after 14 days (0.40±0.04). In BMMSCs group, some acinar and ductal cells showed positive nuclear reaction significantly increased at day 14 (2.36±0.27) more than day 7 (1.81±0.09). In Exosomes group, acinar and ductal cells of this group showed positive nuclear reaction increasing at day 14 (4.0±0.14) more than day 7 (3.51±0.22) and was significantly higher compared to BMMSCs group. Interestingly, as regards PCNA positive expression, an insignificant difference existed between BMMSCs-exosomes and negative control group.

**Fig. 4:**
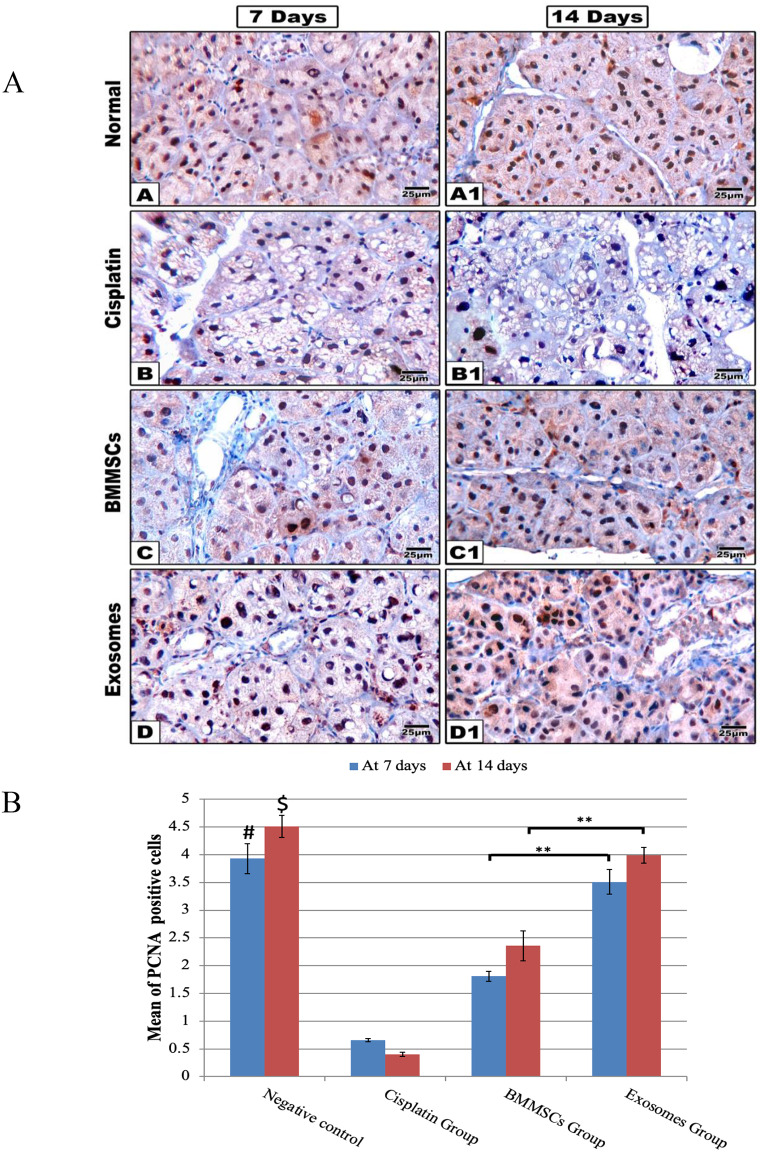
(**A**) Representative histological images of PCNA immune proliferation marker are shown at 7- and 14-days post-treatment. The group received CP (**B, B1**) shows little expression of PCNA immune marker. While PCNA expression markedly increased in BMMSCs (**C, C1**) and the exosomes (**D, D1**) treated groups, scale 100x. (**B**) The Bar chart represents the histomorphometric analysis of PCNA positive expressions at 7- and 14-days post-treatment. Data were presented in means ± SEM. ** *p* < 0.01, # and $ means significant in relation to CP and BMMSCs groups after 7 and 14 days respectively, *** *p* < 0.001

#### Malondialdehyde (MDA) (Fig. 5)

**Fig. 5:**
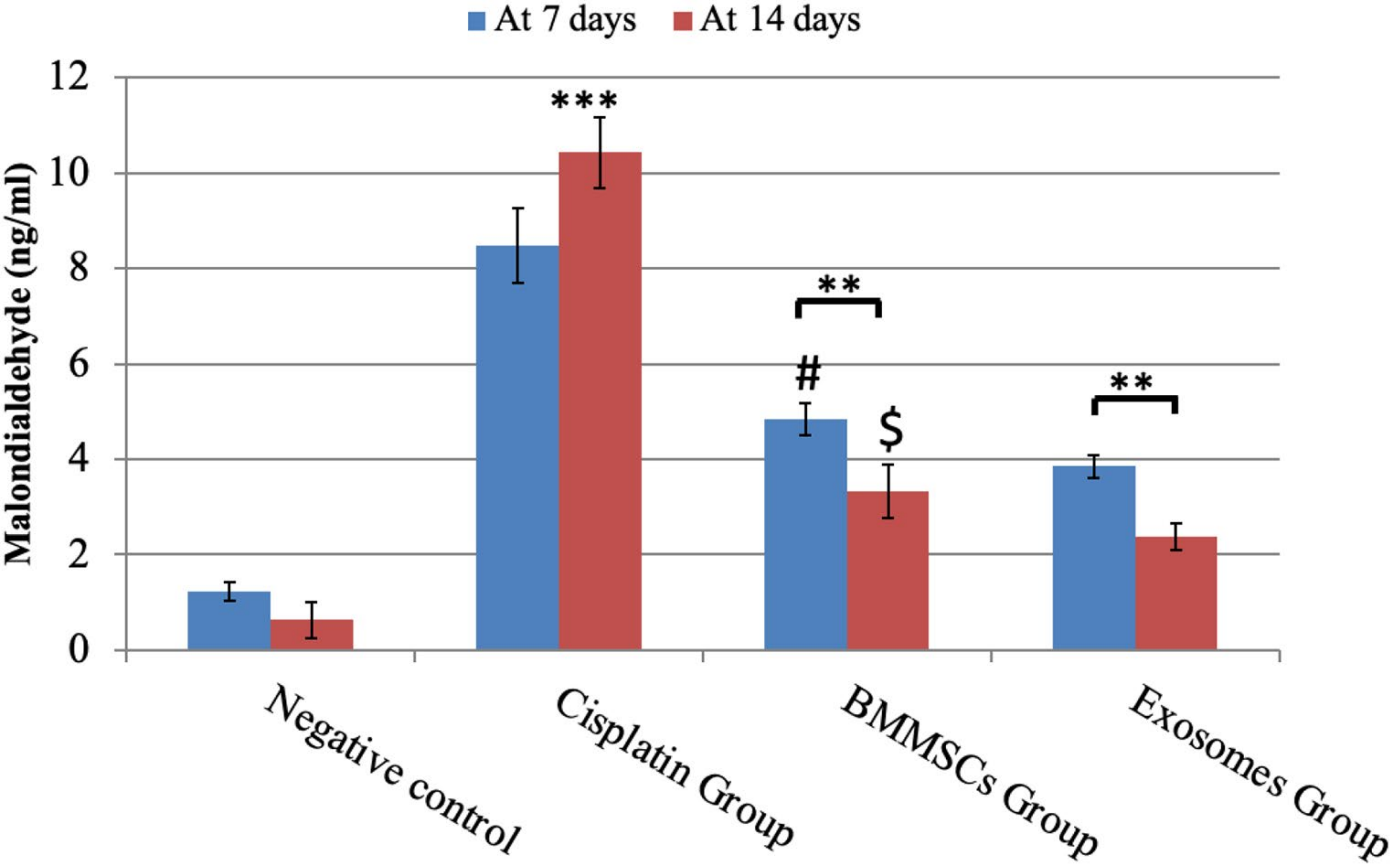
MDA analysis in parotid glands exposed to different treatments for 7 and 14 days. Data were expressed in means ± SDs; *n* = 14. ** P *< 0.01*, *** P *< 0.001.****#*** Means significant to exosomes group after 7 days P *< 0.05.* $ means significant to exosomes group after 14 days P *< 0.05*

This test measures oxidative stress by detecting the amount of lipid peroxidation in tissues. The amount of the MDA in the negative control group was (1.22±0.20) at day 7 and (0.63±0.38) at day 14 in all the tested samples. While in CP group, MDA values were significantly increased than both control and treated groups in all tested time points (after 7 days: 8.49±0.78 and after 14 days: 10.43±0.75) indicating significant increase in lipid peroxidation and OS. The MDA level in BMMSCs group was significantly decreased at day 14 (3.33±0.57) in comparison to what was seen at day 7 (4.85±0.24), while in exosomes treated group, MDA.

was significantly decreased at the two tested time points (after 7 days: 3.85±0.24 and after 14 days: 2.38±0.27) in comparison to BMMSCs treated group suggesting the powerful effect of the derived exosomes in alleviating the lipid peroxidation and OS in comparison to the cell-based therapy.

#### Annexin V (Fig. 6)

**Fig. 6:**
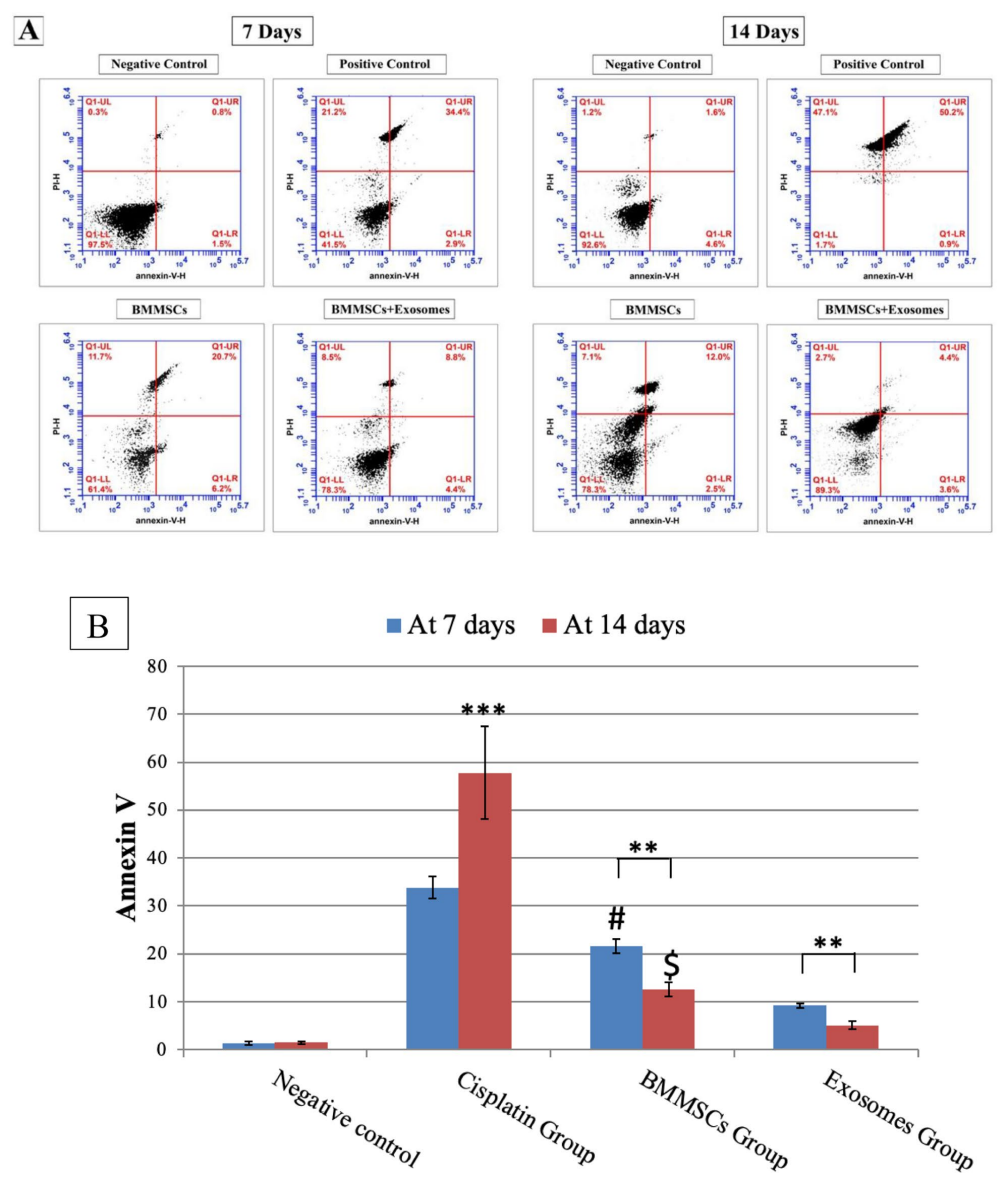
**A**: Showing flow plots for Annexin V (early marker of apoptosis) in parotid salivary gland after 7 and 14 days of treatment analysed by flow cytometry. Apoptosis was measured by Annexin V-FITC/PI staining of live cells. **B**: Demonstrating the percentage of apoptotic cells at 7- and 14-days post-treatment. Data are presented in means ± SDs. ***P < 0.001, **P < 0.01. ***#*** Means significant to exosomes group after 7 days P *< 0.05.* $ means significant to exosomes group after 14 days P *< 0.05*

To determine the effects of BMMSCs and their derived exosomes on parotid salivary gland cells apoptosis, flow cytometry was used to confirm the apoptotic changes. Flow cytometry was used to assess apoptosis in cells stained with Annexin V-FITC/PI. Figure 5 showed the percent of annexin positive cells (control: after 7 days: 1.31±0.36, after 14 days: 1.46±0.20), (CP: after 7 days: 33.80±2.30 and after 14 days: 57.81±9.63). In a time-dependent manner, cisplatin induced cellular apoptosis of salivary gland cells, showing a significant rise of apoptotic cells after 14 days more than after 7 days of treatment. Noteworthy that in BMMSCs and exosomes groups, the percentage of apoptotic cells were significantly reduced in comparison to CP group after 7 and 14 days of treatment (BMMSCs; at 7 days (21.66±1.44) & at 14 days (12.60±1.43), BMMSCs-Exosomes; at 7 days (9.19±0.49) & at 14 days (5.12±0.87). The effect of BMMSCs-exosomes was more significantly pronounced (*P* < 0.01) than the BMMSCs. Notably, the percent of annexin positive cells demonstrated a more significant reduction (*P* < 0.01) in BMMSCs-exosomes group (5.12±0.87) more than BMMSCs group (12.60±1.43) after 14 days of treatment.

## Discussion

CP is a widely used antineoplastic agent that is used for treating various types of cancers in humans. CP is commonly given by the intravenous route and upon its absorption into cancer cells, it interacts with macromolecules in the cell and exerts cytotoxicity via binding to DNA and forming adducts with subsequent suppression of DNA synthesis and cellular division. The main mechanism of action of CP has been linked to its ability to induce apoptosis due to the generation of ROS due to lipid peroxidation [44].

In this research, adult males albino rats were selected, being small in size, easy to be housed, handled, and due to their rapid growth while females were excluded as hormonal changes may influence the outcomes [45]. Due to homing and paracrine properties of MSCs, previous research revealed insignificant differences between systemic and local (intra glandular injection) delivery routes of MSCs for repairing irradiated salivary gland in terms of gland weight, cellular proliferation rate, number of acinar cells and vessels [46].

The process of cell homing or migration, a prerequisite for tissue regeneration, is characterised as a directional movement of cells in response to a chemoattractant and is crucial for the development and maintenance of multicellular organisms [47, 48]. Cell homing, which is a feature of MSCs, is accomplished by a number of crucial biological processes that guarantee efficient transport and localisation in the desired location. This “homing effect”, enables MSCs to reach the injured site and start doing its role in regeneration [49, 50].

Alvarez-Erviti et al. discovered that MSCs-exosomes preserve their mother cells’ homing characteristics [51].

In particular, exosomes contain cyclic adenosine monophosphate (cAMP), which can be actively synthesised and released to promote chemotaxis [52]. Sung and colleagues have reported that migrating cells leave stationary exosome trails to be used as pathways for follower cells [53]. Furthermore, the speed of cell migration is controlled by cell adhesion molecules, such as integrins and fibronectin, which are abundant in exosomes [54]. Exosomes transport membrane-linked matrix metalloproteinases (MMPs) to facilitate the invasive motility of cells across ECM, and proteolytic ECM breakdown greatly facilitates cell migration [55].Thus, in this study, we injected MSCs intravenously being easier and also to prevent the possible local effect of intra glandular injection.

In this study, the histological findings of CP group one-week post-treatment showed that glandular acini were completely reduced, with significant interlobular spaces. This was in agreement with Shaymaa and co-workers who found that treatment with CP was associated with a significant reduction in the size of lobules and acini, interstitial oedema and abundant cytoplasmic vacuoles [56]. In this study parotid glands received CP exhibited nuclear indications of degeneration and nuclear changes, including fragmentation and degeneration of nuclei, and irregular nuclear membranes and variations in size and shape. Intra-cytoplasmic vacuoles were also detected, and this could be because of the release of free radicals from CP leading to damage to cell structure [57]. Terzi and colleagues suggested that vacuoles might be related to significant degeneration and apoptosis of acinus cells triggered by CP treatment [58]. Studies explained the appearance of crescent nuclei by the presence of large vacuoles formed due to hydropic degeneration that displaced nuclei from their original position or as areas of chromatin condensation at the nucleus’s periphery [59, 60]. Earlier studies clarified that the cause is that nuclear chromatin underwent segregation so that the heterochromatin was separated from the euchromatin, causing a crescent form appearance or because of cytoplasmic accumulation of different substances e.g. mucins [61, 62]. Yang et al. also described this nuclear shape as cells actively undergoing apoptosis when they were testing the effect lipopolysaccharide on DPSCs [63]. J. Levi explained the mechanism of action of CP in his study. CP undergoes activation as it enters into the cell and is relocated via water molecules replacing chloride atoms on CP. It Then interacts with any nitrogen donor atom on the nucleic acid, leading to DNA damage, suppression of cell growth, and apoptosis [64].

In comparison to CP treated rats, the BMMSCs- treated rats demonstrated superior histologic outcomes, and there were noticeable decreases in vacuolation of acinar cells and nuclear degeneration. Also, no vacuoles were seen in the acini and ducts. This agreed with Tran and his team who reported improved function of submandibular glands, increased proliferation, inhibition of apoptosis, and improved vascularity upon BMMSCs administration [65]. Our study revealed improvement in the BMMSCs treated rats 2 weeks post-treatment. This was evidenced by the presence of fewer vacuoles; cell boundaries were more distinct; normal appearance of striated duct, with basal striation and abundant patent vessels around it. Similar to the results reported by Lombaert and colleagues, the current study findings indicated that BMMSCs could colonize the damaged salivary gland and stimulate its regeneration through paracrine signalling; thus improving gland function and morphogenesis [66]. The paracrine effect of BMMSCs sheds the light on the role of BMMSCs-exosomes treated group that enhanced the regenerative capabilities of SGs returning the histological architecture into the normal appearance.

The production of paracrine mediators including growth factors and chemokines were found to have chemoprotective effects [67]. Like natural nanoparticles, exosomes have significantly gained attention as a promising cell-free therapy. That could probably suggest that BMMSCs and their exosomes can interact with resident cells in response to tissue injury inducing proliferative factors. This may explain greater number of cells expressed PCNA than what was seen in the PBS group in which a basic PBS formula (with no Ca^+ 2^ or magnesium) was injected to prevent any likely effect of such minerals on healthy cells. Thus, the inhibition of apoptosis induced by BMMSCs-exosomes is possibly linked to the anti-apoptotic activities of BMMSCs because of the local paracrine secretion [68]. Zhang et al., confirmed that BMMSCs-derived bioactive components were capable of reducing gland tissue fibrosis through down-regulation of inflammatory markers and improving the resistance of tissue to OS-induced apoptosis [69].

CP induced apoptosis either via extrinsic or intrinsic pathways (death receptor-dependent pathway or mitochondrial pathway, respectively). The extrinsic pathway is initiated by death receptors’ stimulation, whereas mitochondrial membrane disruption is associated with the release of pro-apoptotic factors (e.g. cytochrome c) into the cytosol, with activation of caspase 9 and then caspase 3 [70]. This explained our finding where the percentage of apoptotic cells were significantly greater in CP treated rats than other treated or control groups. While BMMSCs and their derived exosomes could significantly reduce the percentage of apoptotic cells after CP administration. This could be explained by proteomic profiling of BMMSCs-exosomes revealing the presence of 189 proteins. These proteins have major roles in protective cellular pathways including cell adhesion and inhibition of apoptosis [71]. The expression levels of pro-inflammatory cytokines (TNF-a, IL-1b) and pro-apoptotic protein (Bax) were found to be significantly downregulated in rats following systemic administration of MSCs-exosomes, whereas anti-inflammatory cytokine (IL-10) and anti-apoptotic protein (Bcl-2) were found to be significantly upregulated. This could give explanation for the superior effect of exosomes over cell-based therapy [72].

CP can directly generate ROS or indirectly through mitochondria. CP cytotoxic effects are attributed to lipid peroxidation, which induces OS in the cell membrane, and triggers an apoptotic process [73–75]. In this study, CP treated group showed high levels of MDA in relation to other groups while BMMSCs-exosomes significantly reduced MDA level in comparison to BMMSCs alone. Our findings in this regard were in agreement with previously published studies, demonstrating that CP induces lipid peroxidation, increases MDA generation, and causes DNA damage [76]. OS and changes in axonal transport are classic features of the pathogenetic process of disease [77]. In our study, OS significantly reduced in the groups received BMMSCs-exosomes more than those treated with BMMSCs alone. This could be explained by the presence of certain proteins (e.g. SOD1 and SOD3) in BMMSCs-exosomes that have a role in the response to stress. These proteins have the ability to neutralize free radicals. Other proteins in BMMSCs-exosomes (e.g. cytoskeleton proteins) have a role in cytoplasmic transport, explaining their protective effects on salivary gland cells [71]. Compared to BMMSCs therapy, exosomes exert intense biological effects because of their direct fusion with target cells. Exosomes therapy also improved antioxidant capacities demonstrated by increased ferric ion reducing antioxidant power and glutathione peroxidase or superoxide dismutase activities in oxidative stress-induced cells; it also alleviated cellular and histological responses to inflammation and oxidation in cell or animal models. Hence, BMMSCs-exosomes treatment decreased reactive oxygen species generation, DNA damage and mitochondrial changes [78].

## Conclusion

This study concludes that BMMSCs-exosomes have the potential to remodel chemotherapeutically-damaged parotid salivary gland through cytoprotection of parenchymal elements and proliferation induction. Administration of cell exosomes, without cell transplantation, can provide a therapeutic option for tissue repair in injured salivary glands.

## Data Availability

No datasets were generated or analysed during the current study.
